# Clinicodemographic correlates of psychotic features in bipolar disorder – a multicenter study in China

**DOI:** 10.1186/s12888-023-04761-5

**Published:** 2023-05-24

**Authors:** Zhi-Fang Zhang, Juan Huang, Xue-Quan Zhu, Xin Yu, Hai-Chen Yang, Xiu-Feng Xu, Yi-Ru Fang, Qing-Rong Tan, Hui-Chun Li, Gang Wang, Ling Zhang

**Affiliations:** 1 The National Clinical Research Center for Mental Disorders & Beijing Key Laboratory of Mental Disorders, Beijing Anding Hospital, Capital Medical University, Beijing, China; 2 Peking University Institute of Mental Health (the sixth Hospital) & National Clinical Research Center for Mental Disorders & the key Laboratory of Mental Health, Ministry of Health (Peking University), Beijing, China; 3 Division of Mood Disorders, Shenzhen Mental Health Centre, Guangdong Province Shenzhen, China; 4 Department of Psychiatry, the First Affiliated Hospital of Kunming Medical University, Yunnan Province Kunming, China; 5 Division of Mood Disorders, Shanghai Mental Health Center, Shanghai Jiao Tong University School of Medicine, Shanghai, China; 6 Department of Psychiatry, Xijing Hospital, Fourth Military Medical University, Shaanxi Province, Xi’an, China; 7 The Second Affiliated Hospital, College of Medicine, Zhejiang University, Zhejiang Province Hangzhou, China; 8 Advanced Innovation Center for Human Brain Protection, Capital Medical University, 100069 Beijing, China

**Keywords:** Bipolar disorder, Psychotic symptoms, Multicenter, Univariate analysis, Multivariate analyses

## Abstract

**Background:**

Psychotic symptoms are prevalent in patients with bipolar disorder (BD). However, nearly all previous studies on differences in sociodemographic and clinical factors between patients with (BD P +) and without (BD P-) psychotic symptoms were conducted in Western populations, and limited information is known in China.

**Method:**

A total of 555 patients with BD from seven centers across China were recruited. A standardized procedure was used to collect patients’ sociodemographic and clinical characteristics. The patients were divided into BD P + or BD P- groups based on the presence of lifetime psychotic symptoms. Mann–Whitney U test or chi-square test was used to analyze differences in sociodemographic and clinical factors between patients with BD P + and BD P-. Multiple logistic regression analysis was conducted to explore factors that were independently correlated with psychotic symptoms in BD. All the above analyses were re-conducted after the patients were divided into BD I and BD II group according to their types of diagnosis.

**Results:**

A total of 35 patients refused to participate, and the remaining 520 patients were included in the analyses. Compared with patients with BD P-, those with BD P + were more likely to be diagnosed with BD I and mania/hypomania/mixed polarity in the first mood episode. Moreover, they were more likely to be misdiagnosed as schizophrenia than major depressive disorder, were hospitalized more often, used antidepressants less frequently, and used more antipsychotics and mood stabilizers. Multivariate analyses revealed that diagnosis of BD I, more frequent misdiagnosis as schizophrenia and other mental disorders, less frequent misdiagnosis as major depressive disorder, more frequent lifetime suicidal behavior, more frequent hospitalizations, less frequent use of antidepressants, more frequent use of antipsychotics and mood stabilizers were independently correlated with psychotic symptoms in BD. After dividing the patients into BD I and BD II groups, we observed notable differences in sociodemographic and clinical factors, as well as clinicodemographic correlates of psychotic features between the two groups.

**Conclusions:**

Differences in clinical factors between patients with BD P + and BD P- showed cross-cultural consistency, but results on the clinicodemographic correlates of psychotic features were not. Notable differences between patients with BD I and BD II were found. Future work exploring the psychotic features of BD needs to take types of diagnosis and cultural differences into consideration.

**Trial registration:**

This study was first registered on the website of the ClinicalTrials.gov (https://clinicaltrials.gov/) on 18/01/2013. Its registration number is NCT01770704.

**Supplementary Information:**

The online version contains supplementary material available at 10.1186/s12888-023-04761-5.

## Introduction

Bipolar disorder (BD) is a serious, chronic, and debilitating mental illness. It is characterized by recurrent mood states of mania/hypomania, depression, and euthymia. Psychotic symptoms, such as hallucinations and delusions, are common clinical symptoms in BD [1, 2] and often occur during manic/hypomanic and depressive mood states [2, 3]. More than 50% of patients with BD will experience psychotic symptoms at least once during their lifetime [3–5].

Differences in sociodemographic and clinical features between patients with (BD P +) and without (BD P-) lifetime psychotic symptoms have been frequently reported. Compared with patients with BD P-, patients with BD P + show greater symptom severity [6], worse prognosis [5, 7], decreased rates of recovery [8, 9], shorter times to first recurrence [10, 11], more frequent mood episodes and hospitalizations [12, 13], increased risk of relapses [14], decreased response to lithium [15], and earlier age at onset of disease [16, 17]. Patients with BD P + are also more likely to be diagnosed with BD I and more likely to be misdiagnosed as schizophrenia [18], exhibit more manic/hypomanic/mixed episode in the first and the most recent episode, higher comorbidity of alcohol/substance use disorder, and higher rates of involuntary admission; present more psychosocial rehabilitation history, lower rates of comorbid anxiety disorders, poorer social functioning, shorter duration of most recent episode, increased use of antipsychotics, and decreased use of antidepressants [19–21].

However, previous studies investigating the clinicodemographic correlates of psychotic features in BD have reported diverse findings. For instance, a study that recruited only patients with BD I found that psychotic symptoms were associated with an earlier onset of disease and more frequent hospitalizations [17], while another study reported that psychotic symptoms were associated with younger age, less likelihood of comorbidity with agoraphobia, and higher likelihood of comorbidity with alcohol abuse [22]. However, studies that recruited both patients with BD I and BD II found that psychotic symptoms were associated with a higher frequency of a first diagnosis different from BD or major depressive disorder (MDD), a prevalent manic polarity, and a higher number of lifetime manic episodes (more than six) [23]. Given that previous studies have frequently reported differences in clinicodemographic factors, including psychotic features between patients with BD I and BD II [24, 25], it is possible that patients’ different types of diagnosis may contribute to the inconsistent results.

We noticed that all the above research findings on patients with BD P + and BD P- were obtained in Western populations. However, numerous biological and sociocultural factors may contribute to differences in the prevalence and clinical characteristics of mood disorders in different parts of the world [26, 27]. Thus, findings acquired in Western populations may not cover the full range of clinical characteristics of Chinese patients [28], and certain differences may exist [29, 30]. Moreover, the Chinese Human Connectome Project (CHCP) recently confirmed that there were some similarities and differences between Chinese Han populations and Western populations in brain function and brain structure [31]. Considering that BD is an illness of the brain, it is expected that there may be some similarities and differences between patients with BD recruited from Chinese Han populations and Western populations. To the best of our knowledge, only one study recruited Chinese patients and reported sociodemographic and clinical differences between patients with BD P + and BD P- [32]. Patients with BD P + were younger and had earlier age of disease onset; however, participants were all recruited from a single center, and data on sociodemographic and clinical factors were few, thereby limiting the representation when we consider the characteristics of BD I in a wider Chinese population.

This study aimed to analyze differences in sociodemographic and clinical information between patients with BD P + and BD P- and explore the correlates of psychotic symptoms in BD, including both BD I and BD II. Data were obtained from our previous multicenter studies [29, 30]. The results may identify possible contributing factors to psychotic symptoms in a broader BD population, especially in the Chinese population.

## Materials and methods

### Subjects

This cross-sectional, multicenter study was initiated by the Chinese Society of Psychiatry and conducted from January 2013 to January 2014 (registration number: NCT01770704, First registration date: 18/01/2013). In total, 555 inpatients or outpatients with BD were recruited. Of these, 35 (6.3%) patients refused, and 520 (93.7%) patients completed the entire research process. The patients were from seven centers, including tertiary psychiatric hospitals and the psychiatry department of general hospitals, which were distributed in the southern, northern, western, eastern, and central parts of China. All patients met the diagnostic criteria of BD I or BD II in accordance with Diagnostic and Statistical Manual of Mental Disorders-IV and had experienced at least one depressive or manic episode within the past 12 months. The study protocol was approved by the Clinical Research Ethics Committee of all seven centers. Written informed consent was obtained from all patients before they were allowed to participate in this study.

### Assessment procedure

The detailed assessment procedure was described in our previous studies [29, 30]. Simply put, all patients were receiving treatment in the participating hospitals/units and were referred by their treating psychiatrists to the investigators. Once they met our entry criteria, they were invited to participate in this study. Then, their basic demographic and clinical data were collected using a standard data collection form that was devised for this study. In this clinical interview, the assessment of lifetime psychotic symptoms was obtained from previous medical records or evaluated through psychiatric interviews by experienced psychiatrists. If the patient experienced any psychotic symptom, including hallucination or delusion during the process of any previous episode, he or she was assigned to the BD P + group; otherwise, he or she was assigned to the BD P- group.

### Statistical analyses

Data analyses were performed using SPSS 20.0 software. For continuous variables, one-sample Kolmogorov–Smirnov test was used to detect the normality of data distribution. Differences in sociodemographic and clinical characteristics between patients with and without lifetime psychotic symptoms were analyzed using Mann–Whitney U test or chi-square test. All variables were used as independent variables in the following multiple logistic regression analysis. Psychotic symptoms were the dependent variable, and the “Enter” method was used. The significance level was set at *P* < 0.05 (two-tailed). Then, the patients with BD were divided into BD I and BD II subgroups based on their diagnosis types, and subsequently, all the aforementioned univariate and multivariate analyses were repeated for each subgroup.

## Results

Of the 520 participants with BD, 217 patients experienced psychotic symptoms in their lifetime. Table 1 presents the descriptive statistics for patients with or without lifetime psychotic symptoms and for the entire sample. Compared with the BD P- group, the BD P + group showed a higher percentage in several aspects, including earlier age of disease onset, diagnosis of BD I, and mania/hypomania/mixed episode type in the first mood episode. Patients with BD P + showed fewer previous misdiagnoses of major depressive disorder, more previous misdiagnoses of schizophrenia, more psychiatric hospitalization history, more frequent use of antipsychotics, mood stabilizers, or two or more kinds of mood stabilizers, and less frequent use of antidepressants. Multiple logistic regression analysis revealed that diagnosis of BD I, more frequent misdiagnosis as schizophrenia and other mental disorders, less frequent misdiagnosis as major depressive disorder, more frequent hospitalizations, more frequent lifetime suicidal behavior, less frequent use of antidepressant, more frequent use of antipsychotics and mood stabilizers were independently correlated with psychotic symptoms in BD (Table 2).

**Table 1:** Socio-demographic and clinical characteristics of the patients with or without psychotic symptoms

	Total sample (*N* = 520)	BD P- (*N* = 303)	BD P + (*N* = 217)	Z/χ^2^	*P*
Age (Mean ± SD) ^a^	35.15 ± 13.22	35.57 ± 13.69	34.55 ± 12.55	-0.59	0.558
Male (N, %) ^b^	252(48.46%)	147(48.51%)	105(48.39%)	0.001	0.977
Years of education (Mean ± SD) ^a^	13.10 ± 3.40	12.99 ± 3.44	13.25 ± 3.34	-0.39	0.696
Employed (N, %) ^b^	290(55.77%)	171(56.44%)	119(54.84%)	0.13	0.718
Living with family (N, %) ^b^	478(91.92%)	274(90.43%)	204(94.01%)	2.18	0.140
Comorbid substance abuse (N, %) ^b^	36(6.92%)	18(5.94%)	18(8.29%)	1.09	0.297
Age of disease onset (N, %) ^b,c^				4.81	**0.028**
Early onset (≤ 25)	250(48.08%)	145(47.85%)	125(57.60%)		
Late onset (> 25)	270(51.92%)	158(52.15%)	92(42.40%)		
Duration of undiagnosed bipolar disorder (Month, Median and quartiles) ^a^	10(0.00, 54.19)	12(0.00, 49.25)	9.75(0.00, 64.50)	-0.14	0.888
Types of bipolar disorder				49.69	** < 0.001**
BD I (%) ^b^	399(76.73%)	199(65.68%)	200(92.17%)		
BD II (%) ^b^	121(23.27%)	104(34.32%)	17(7.83%)		
Duration of illness (Month, Median and quartiles)^a^	45.00(13.00, 111.00)	42.00(12.75, 93.50)	48.00(13.00, 132.50)	-1.56	0.119
Polarity of first mood episode (N, %) ^b^				25.85	** < 0.001**
Depressive	307(59.04%)	207(68.32%)	100(46.08%)		
Manic/hypomanic/mixed	213(40.96%)	96(31.68%)	117(53.92%)		
History of misdiagnosis					
Major depressive disorder (N, %)^a^	270(51.92%)	193(63.70%)	77(35.48%)	40.32	** < 0.001**
Schizophrenia (N, %)^a^	89(17.12%)	12(3.96%)	77(35.48%)	88.57	** < 0.001**
Other mental disorders (N, %)^a^	89(17.12%)	44(14.52%)	45(20.74%)	3.44	0.063
Family history of mental disorders (N, %)^b^	151(29.04%)	91(30.03%)	60(27.65%)	0.35	0.555
History of psychiatric hospitalization (N, %)^b^	415(79.81%)	218(71.95%)	197(90.78%)	27.84	** < 0.001**
Lifetime suicide behavior (N, %)^b^	54(10.38%)	28(9.24%)	26(11.98%)	1.02	0.312
Received drug treatment within the past 12 months	487(93.65%)	281(92.74%)	206(94.93%)	1.02	0.312
Antidepressants use (N, %)^b^	160(30.77%)	123(40.59%)	37(17.05%)	32.90	** < 0.001**
Antipsychotics use (N, %)^b^	392(75.38%)	201(66.34%)	191(88.02%)	32.03	** < 0.001**
Mood stabilizer use (N, %)^b^	439(84.42%)	246(81.19%)	193(88.94%)	5.78	**0.016**
Two or more mood stabilizers use (N, %)^b^	119(22.88%)	58(19.14%)	61(28.11%)	5.76	**0.016**
Received non-drug treatment within the past 12 months (N, %)^b^	61(11.73%)	33(10.89%)	28(12.90%)	0.49	0.482
Combined with chronic diseases requiring long-term treatment (N, %)^b^	58(11.15%)	29(9.57%)	29(13.36%)	1.84	0.175

BD P-: The patients who were without psychotic symptoms; BD P + : The patients who were with psychotic symptoms

^a^ These variables were compared by using Mann–Whitney U test

^b^ These variables were compared by using chi-square test

^c^ Following previous studies [41, 42], age of 25 years was used as the cutoff point to define early onset and late onset

**Table 2:** Multivariate logistic regression analysis of bipolar disorder with psychotic symptoms

Independent variables	Beta	SE	Wald's test	*P*	Odds ratio	95% Confidence interval	
						Lower limit	Higher limit
Age	0.01	0.01	0.64	0.423	1.01	0.98	1.04
Male	-0.40	0.23	2.94	0.086	0.67	0.43	1.06
Years of education	0.05	0.04	1.70	0.192	1.05	0.98	1.13
Unemployment	-0.23	0.24	0.88	0.348	0.80	0.49	1.28
Living alone	-0.72	0.46	2.45	0.118	0.49	0.20	1.20
Early onset	0.22	0.33	0.43	0.515	1.24	0.65	2.37
Diagnosis of BD I	1.08	0.34	9.99	**0.002**	2.94	1.51	5.74
Duration of illness > 60 months^a^	-0.05	0.29	0.03	0.864	0.95	0.54	1.68
Polarity of first mood episode was manic/hypomanic/mixed	-0.03	0.26	0.01	0.906	0.97	0.58	1.62
History of misdiagnosed as major depressive disorder	-0.55	0.27	4.20	**0.040**	0.58	0.34	0.98
History of misdiagnosed as schizophrenia	2.57	0.39	44.47	** < 0.001**	13.06	6.14	27.79
History of misdiagnosed as other mental disorders	0.65	0.29	4.91	**0.027**	1.91	1.08	3.39
Family history of mental disorders	0.10	0.26	0.16	0.693	1.11	0.66	1.86
History of psychiatric hospitalization	0.77	0.33	5.35	**0.021**	2.16	1.13	4.16
Lifetime suicide behavior	0.87	0.37	5.58	**0.018**	2.38	1.16	4.87
Received drug treatment within the past 12 months	-0.89	0.71	1.58	0.208	0.41	0.10	1.64
Antidepressants use	-0.65	0.29	5.02	**0.025**	0.52	0.30	0.92
Antipsychotics use	0.94	0.35	7.43	**0.006**	2.57	1.30	5.07
Mood stabilizer use	0.94	0.45	4.42	**0.035**	2.56	1.07	6.16
Two or more mood stabilizers use	-0.50	0.32	2.50	0.114	0.61	0.32	1.13
Received non-drug treatment within the past 12 months	0.36	0.34	1.12	0.290	1.43	0.74	2.80
Combined with chronic diseases requiring long-term treatment	0.47	0.37	1.62	0.203	1.60	0.78	3.28

^a^ Following previous studies [35], 60 months (i.e. 5 years) was used as the cutoff point to define short- and long- duration illness

After dividing the patients with BD into BD I and BD II groups, our analysis revealed distinct clinical correlates of psychotic symptoms in each group. In the BD I group, patients with psychotic symptoms were more likely to have experienced mania/hypomania/mixed episodes in their first mood episode, fewer misdiagnoses of major depressive disorder, more frequent misdiagnoses of schizophrenia and other mental disorders, more frequent psychiatric hospitalizations, and greater use of antipsychotics, mood stabilizers, or multiple mood stabilizers, and less frequent use of antidepressants (Table S1). Furthermore, multiple logistic regression analysis indicated that the independent predictors of psychotic symptoms in BD I were more frequent misdiagnosis as schizophrenia and other mental disorders, more frequent hospitalizations, more frequent lifetime suicidal behavior, less frequent use of antidepressants, and more frequent use of antipsychotics (Table S2).

In contrast, in the BD II group, patients with psychotic symptoms were more likely to be unemployed and have chronic diseases requiring long-term treatment. They also reported more previous misdiagnoses of schizophrenia and greater use of antipsychotics (Table S3). Multiple logistic regression analysis indicated that the independent predictor of psychotic symptoms in BD II was the presence of chronic diseases requiring long-term treatment (Table S4).

## Discussion

This multicenter study was conducted in seven major psychiatric hospitals/units across China. Numerous differences were found in sociodemographic and clinical characteristics between patients with BD P + and BD P-. Patients with BD P + had higher rates of diagnosis of BD I, previous misdiagnosis as schizophrenia, history of psychiatric hospitalization, and more antipsychotic and mood stabilizer use within the past 12 months. Moreover, they had lower rates of previous misdiagnosis as MDD and antidepressant use within the past 12 months. These factors were independently correlated with psychiatric symptoms in BD. Besides, after dividing the patients into BD I and BD II groups, we observed significant differences in the sociodemographic and clinical factors, as well as the clinicodemographic correlates of psychotic features between the two groups.

In the total sample of BD, consistent with the findings of previous studies on Western populations, the current research revealed that patients with BD P + in China had earlier age of disease onset [16, 17], exhibited more diagnosed as BD I, more previously misdiagnosed as schizophrenia [18], more elevated (manic/hypomanic/mixed) polarity of the first mood episode, more antipsychotics use, fewer antidepressants use [19, 20], and more frequent hospitalization (i.e., their symptoms were more severe) [12, 13]. Compared with patients with BD P-, those with BD P + had fewer previous misdiagnoses of MDD before they were correctly diagnosed with BD, which may be related to two reasons. First, the majority of patients with BD P + were diagnosed with BD I, which means that a definite history of a manic episode eases the diagnosis of BD; second, the existence of psychotic symptoms brings more similarities between BD and schizophrenia [18], thereby increasing the rates of misdiagnosis as schizophrenia but not MDD. Moreover, patients with BD P + were more likely to be treated with one or two or more kinds of mood stabilizers. The possible reasons may include the following: (1) mood stabilizers are always the main drug prescription in the treatment of mania or mixed episodes according to the Chinese BD treatment guidelines [36]; (2) up to 88% of patients with BD experience psychotic symptoms during manic mood states [5], whereas the proportion reaches 45% during depressive mood states [37]; psychotic symptoms are prone to correlate with manic mood states and then increase the rate of mood stabilizer use; (3) patients with BD P + often have a decreased response to mood stabilizers, such as lithium [15], which results in the use of another or more mood stabilizers as combination treatment [13]. All these findings depicted an overview of the sociodemographic and clinical characteristics of patients with psychotic BD in China.

More importantly, we observed that several factors were independently correlated with psychotic symptoms in BD. Diagnosis of BD I, psychiatric hospitalization history, previous misdiagnosis as schizophrenia and other mental disorders, previous suicidal behavior, and antipsychotic and mood stabilizer use were positively correlated with psychotic symptoms. Psychotic symptoms can be observed in approximately 70% of patients with BD I [5, 17, 38] and in 20% of patients with BD II [13]. In the current study, the proportion of BD P + in BD I was 50.1% (200/399) and that in BD II was 14.0% (17/121). Despite the sample bias, the gap between the two groups remained significant. Furthermore, psychotic symptoms can explain the increased rates of misdiagnosis of schizophrenia and antipsychotic use [19, 39]. The existence of lifetime psychiatric comorbidity can explain the correlation of psychotic symptoms with misdiagnosis as other mental disorders [22]. Meanwhile, psychotic symptoms usually indicate a more severe or urgent clinical status, resulting in hospitalization for intensive care [40]. Patients with BD P + responded better to lithium monotherapy than the BD P- group [13], resulting in more mood stabilizer use. The association of psychotic BD with a high risk of suicide has been frequently reported in previous studies [41], and this study further confirmed the relationship of suicidal behavior with psychotic symptoms in BD. In addition, the results demonstrated that previous misdiagnosis of MDD and antidepressant use were negatively correlated with psychotic symptoms. Antidepressants are not commonly used in clinical practice for patients with psychotic BD [19, 20] because they are often diagnosed as BD I. This phenomenon is also in line with the lower rates of MDD misdiagnosis. Moreover, we noticed that previous misdiagnosis as other mental disorders and previous suicidal behavior were not significant variables in univariate analyses but became significant in the multivariable analysis. This suggested that the intergroup differences cannot fully reflect possible interactions among these variables, and multivariable analysis may be a supplementary method to discover complex correlations among different variables. In brief, these variables are important contributing factors or outcomes of psychotic symptoms.

In addition, when comparing our results with those found in another site in China [32], we found that age may be a variable susceptible to sample bias. That is, when recruiting subjects from different hospitals/sites, their age distribution may be different. From this point of view, the current study may be more convincing in detecting age differences between patients with BD P + and BD P- because we recruited patients from seven centers that were distributed in the southern, northern, western, eastern, and central parts of China. Therefore, we believe that there is no age difference between patients with BD P + and BD P- is the most realistic result. Different from age, age of disease onset may not be susceptible to sample bias but shows cross-site and cross-cultural consistency. In other words, all patients with BD P + from different sites in China or Western countries showed a higher percentage of an earlier age of disease onset.

After dividing the patients with BD into BD I and BD II groups, our results showed significant differences in sociodemographic and clinical factors, as well as clinicodemographic correlates of psychotic features between patients with BD P + and BD P- in each sub-group. This finding is in line with previous studies that have reported differences between BD I and BD II patients [42, 43]. Specifically, in patients with BD I, our results were consistent with previous studies regarding the sociodemographic and clinical factors associated with psychotic symptoms [17, 19]. However, for patients with BD II, the variables included in previous studies were different, making it difficult to draw definitive conclusions [12]. Regarding clinicodemographic correlates of psychotic features, previous studies on Western patients with BD I have yielded inconsistent results [17, 22]. Our study found that one variable (i.e., more frequent hospitalizations) was consistent with a previous multicenter study [17], but other variables not included in previous studies were also identified. It remains unclear whether clinicodemographic correlates of psychotic features in BD II patients show cross-cultural consistency. More studies should be conducted to verify possible clinicodemographic factors correlating with psychotic features in BD, and a multicenter study is recommended.

This study has some limitations. First, all sociodemographic and clinical features were collected retrospectively, which possible resulted in a potential recall bias. Second, our results cannot reflect the treatment profiles of the whole course because patients’ previous treatment history was recorded only within 12 months before their recent admission. However, the longer the patients recalled previous occurrences, the greater the impact of recall bias. Moreover, 12 months seemed to be a common choice in other studies [17]. Third, mood stabilizers recorded in the study were lithium, sodium valproate, carbamazepine, and lamotrigine; second-generation antipsychotics (SGAs) with certain mood-stabilizing properties were excluded. The real purpose of SGA prescription is hard to determine in a retrospective study. Fourth, the sample size of patients with BD P + in the BD II group was small (*N* = 17), which limits the generalizability of our findings. Therefore, future studies should aim to recruit a larger sample size to validate our results.

In conclusion, this multicenter study discovered several correlated factors in sociodemographic and clinical characteristics of psychotic symptoms in patients with BD in China, and most of them were consistent with the findings on Western populations. Moreover, notable differences between patients with BD I and BD II were found. The results expand our understanding of the importance of psychotic symptoms in BD, but further research is needed to explore their features.

## Supplementary Information

**Additional file 1:** **Table S1.** Socio-demographic and clinical characteristics of the patients with BD I with or without psychotic symptoms. **Table S2.** Multivariate logistic regression analysis of bipolar I disorder with psychotic symptoms. **Table S3.** Socio-demographic and clinical characteristics of the patients with BD II with or without psychotic symptoms. **Table S4.** Multivariate logistic regression analysis of bipolar II disorder with psychotic symptoms.

## Data Availability

The datasets used and/or analyzed during the current study are available from the corresponding author on reasonable request.
