# Supplementary material for: Clinicodemographic correlates of psychotic features in bipolar disorder – a multicenter study in China
Source: BMC Psychiatry. 2023 May 24;23:365. doi: 10.1186/s12888-023-04761-5 (PMC10210479; doi:10.1186/s12888-023-04761-5)
Supplement: Supplementary file 1 — Additional file 1: Table S1. Socio-demographic and clinical characteristics of the patients with BD I with or without psychotic symptoms. Table S2. Multivariate logistic regression analysis of bipolar I disorder with psychotic symptoms. Table S3. Socio-demographic and clinical characteristics of the patients with BD II with or without psychotic symptoms. Table S4. Multivariate logistic regression analysis of bipolar II disorder with psychotic symptoms. [file 12888_2023_4761_MOESM1_ESM.docx]

**Supplementary materials**

**Clinicodemographic correlates of psychotic features in bipolar disorder – A multicenter study in China**

Zhi-Fang Zhang^1^, Juan Huang^1^, Xue-Quan Zhu^1^, Xin Yu^2^, Hai-Chen Yang^3^, Xiu-Feng Xu^4^, Yi-Ru Fang^5^, Qing-Rong Tan^6^, Hui-Chun Li^7^, Gang Wang^1,8^, Ling Zhang^1,8,*^

^1^The National Clinical Research Center for Mental Disorders & Beijing Key Laboratory of Mental Disorders, Beijing Anding Hospital, Capital Medical University, Beijing, China

^2^Peking University Institute of Mental Health (the sixth Hospital) & National Clinical Research Center for Mental Disorders & the key Laboratory of Mental Health, Ministry of Health (Peking University), Beijing, China

^3^Division of Mood Disorders, Shenzhen Mental Health Centre, Shenzhen, Guangdong province, China

^4^Department of Psychiatry, the First Affiliated Hospital of Kunming Medical University, Kunming, Yunnan province, China

^5^Division of Mood Disorders, Shanghai Mental Health Center, Shanghai Jiao Tong University School of Medicine, Shanghai, China

^6^Department of Psychiatry, Xijing Hospital, Fourth Military Medical University, Xi’an, Shaanxi province, China

^7^The Second Affiliated Hospital, College of Medicine, Zhejiang University, Hangzhou, Zhejiang province, China

^8^Advanced Innovation Center for Human Brain Protection, Capital Medical University, Beijing, China

^*^Correspondence to: Ling Zhang, The National Clinical Research Center for Mental Disorders & Beijing Key Laboratory of Mental Disorders, Beijing Anding Hospital, Capital Medical University, Beijing, China; Advanced Innovation Center for Human Brain Protection, Capital Medical University, Beijing, China. E-mail: zhangling@ccmu.edu.cn

Table S1. Socio-demographic and clinical characteristics of the patients with BD I with or without psychotic symptoms.

|  | Total sample (N=399) | BD P-  (N=199) | BD P+  (N=200) | Z/χ^2^ | *P* |
| --- | --- | --- | --- | --- | --- |
| Age (Mean±SD) ^a^ | 34.69±13.06 | 35.11±13.80 | 34.29±12.30 | -0.19 | 0.850 |
| Male (N, %) ^b^ | 198(49.62%) | 98(49.25%) | 100(50.00%) | 0.02 | 0.880 |
| Years of education (Mean±SD) ^a^ | 13.22±3.23 | 13.20±3.15 | 13.24±3.31 | -0.06 | 0.949 |
| Employed (N, %) ^b^ | 223(55.89%) | 109(54.77%) | 114(57.00%) | 0.20 | 0.654 |
| Living with family (N, %) ^b^ | 368(92.23%) | 181(90.95%) | 187(93.50%) | 0.90 | 0.340 |
| Comorbid substance abuse (N, %) ^b^ | 30(7.52%) | 14(7.04%) | 16(8.00%) | 0.13 | 0.715 |
| Age of disease onset (N, %) ^b,c^ |  |  |  | 1.57 | 0.211 |
| Early onset (≤ 25) | 217(54.39%) | 102(51.26%) | 115(57.50%) |  |  |
| Late onset (> 25) | 182(45.61%) | 97(48.74%) | 85(42.50%) |  |  |
| Duration of undiagnosed bipolar disorder (Month, Median and quartiles) ^a^ | 6.75(0.00, 49.00) | 6.5(0.00, 39.50) | 7.38(0.00, 57.75) | -1.28 | 0.201 |
| Duration of illness (Month, Median and quartiles)^a^ | 45.00(13.75, 114.50) | 42.50(14.00, 86.25) | 47.50(12.00, 130.75) | -0.97 | 0.332 |
| Polarity of first mood episode (N, %) ^b^ |  |  |  | 10.59 | **0.001** |
| Depressive | 198(49.62%) | 115(57.79%) | 83(41.50%) |  |  |
| Manic/hypomanic/mixed | 201(50.38%) | 84(42.21%) | 117(58.50%) |  |  |
| History of misdiagnosis |  |  |  |  |  |
| Major depressive disorder (N, %)^a^ | 172(43.11%) | 107(53.77%) | 65(32.50%) | 18.40 | **<0.001** |
| Schizophrenia (N, %)^a^ | 85(21.30%) | 11(5.53%) | 74(37.00%) | 58.93 | **<0.001** |
| Other mental disorders (N, %)^a^ | 70(17.54%) | 27(13.57%) | 43(21.50%) | 4.34 | **0.037** |
| Family history of mental disorders (N, %)^b^ | 110(27.57%) | 53(26.63%) | 57(28.50%) | 0.17 | 0.677 |
| History of psychiatric hospitalization (N, %)^b^ | 337(84.46%) | 154(77.39%) | 183(91.50%) | 15.14 | **<0.001** |
| Lifetime suicide behavior (N, %)^b^ | 39(9.77%) | 16(8.04%) | 23(11.50%) | 1.35 | 0.245 |
| Received drug treatment within the past 12 months | 371(92.98%) | 182(91.46%) | 189(94.50%) | 1.42 | 0.234 |
| Antidepressants use (N, %)^b^ | 94(23.56%) | 63(31.66%) | 31(15.50%) | 14.46 | **<0.001** |
| Antipsychotics use (N, %)^b^ | 315(78.95%) | 140(70.35%) | 175(87.94%) | 17.65 | **<0.001** |
| Mood stabilizer use (N, %)^b^ | 339(84.96%) | 162(81.41%) | 177(88.50%) | 3.93 | **0.047** |
| Two or more mood stabilizers use (N, %)^b^ | 96(24.06%) | 39(19.60%) | 57(28.50%) | 4.33 | **0.038** |
| Received non-drug treatment within the past 12 months (N, %)^b^ | 43(10.78%) | 20(10.05%) | 23(11.50%) | 0.22 | 0.641 |
| Combined with chronic diseases requiring long-term treatment (N, %)^b^ | 46(11.53%) | 22(11.06%) | 24(12.00%) | 0.09 | 0.768 |

Note: BD P-: The patients who were without psychotic symptoms; BD P+: The patients who were with psychotic symptoms

^a^ These variables were compared by using Mann-Whitney U test

^b^ These variables were compared by using chi-square test

^c^ Following previous studies ^[38, 39]^, ﻿age of 25 years was used as the cutoff point to define early onset and late onset

Table S2. Multivariate logistic regression analysis of bipolar I disorder with psychotic symptoms.

| Independent variables | Beta | SE | Wald's test | *P* | ﻿Odds ratio | 95% Confidence interval | |
| --- | --- | --- | --- | --- | --- | --- | --- |
|  |  |  |  |  |  | Lower limit | Higher limit |
| Age | 0.01 | 0.02 | 0.16 | 0.687 | 1.01 | 0.98 | 1.04 |
| Male | -0.40 | 0.25 | 2.07 | 0.151 | 0.70 | 0.43 | 1.14 |
| Years of education | 0.02 | 0.04 | 0.29 | 0.593 | 1.02 | 0.94 | 1.11 |
| Unemployment | -0.44 | 0.27 | 2.71 | 0.100 | 0.65 | 0.38 | 1.09 |
| Living alone | -0.64 | 0.49 | 1.65 | 0.199 | 0.53 | 0.20 | 1.40 |
| Early onset | 0.09 | 0.36 | 0.07 | 0.794 | 1.10 | 0.54 | 2.23 |
| Duration of illness > 60 months^a^ | -0.19 | 0.31 | 0.38 | 0.539 | 0.83 | 0.45 | 1.52 |
| Polarity of first mood episode was manic/hypomanic/mixed | 0.08 | 0.28 | 0.08 | 0.775 | 1.08 | 0.63 | 1.86 |
| History of misdiagnosed as major depressive disorder | -0.49 | 0.29 | 2.75 | 0.097 | 0.62 | 0.35 | 1.09 |
| History of misdiagnosed as schizophrenia | 2.65 | 0.41 | 41.22 | **< 0.001** | 14.08 | 6.28 | 31.58 |
| History of misdiagnosed as other mental disorders | 0.88 | 0.33 | 7.30 | **0.007** | 2.41 | 1.27 | 4.57 |
| Family history of mental disorders | 0.34 | 0.29 | 1.32 | 0.251 | 1.40 | 0.79 | 2.49 |
| History of psychiatric hospitalization | 0.92 | 0.37 | 6.07 | **0.014** | 2.51 | 1.21 | 5.20 |
| Lifetime suicide behavior | 1.00 | 0.42 | 5.74 | **0.017** | 2.71 | 1.20 | 6.14 |
| Received drug treatment within the past 12 months | -0.76 | 0.76 | 1.02 | 0.313 | 0.47 | 0.11 | 2.05 |
| Antidepressants use | -0.67 | 0.33 | 4.22 | **0.040** | 0.51 | 0.27 | 0.97 |
| Antipsychotics use | 0.78 | 0.38 | 4.23 | **0.040** | 2.17 | 1.04 | 4.54 |
| Mood stabilizer use | 0.90 | 0.49 | 3.38 | 0.066 | 2.45 | 0.94 | 6.38 |
| Two or more mood stabilizers use | -0.52 | 0.36 | 2.15 | 0.142 | 0.59 | 0.30 | 1.19 |
| Received non-drug treatment within the past 12 months | 0.20 | 0.38 | 0.27 | 0.602 | 1.22 | 0.58 | 2.57 |
| Combined with chronic diseases requiring long-term treatment | 0.30 | 0.41 | 0.55 | 0.457 | 1.36 | 0.61 | 3.02 |

^a^ Following previous studies^[40]^, 60 months (i.e. 5 years) was used as the cutoff point to define short- and long- duration illness

Table S3. Socio-demographic and clinical characteristics of the patients with BD II with or without psychotic symptoms.

|  | Total sample (N=121) | BD P-  (N=104) | BD P+  (N=17) | Z/χ^2^ | *P* |
| --- | --- | --- | --- | --- | --- |
| Age (Mean±SD) ^a^ | 36.64±13.69 | 36.47±13.49 | 37.65±15.24 | -0.16 | 0.875 |
| Male (N, %) ^b^ | 54(44.63%) | 49(47.12%) | 5(29.41%) | 1.85 | 0.173 |
| Years of education (Mean±SD) ^a^ | 12.69±3.90 | 12.58±3.94 | 13.35±3.71 | -0.52 | 0.603 |
| Employed (N, %) ^b^ | 67(55.37%) | 62(59.62%) | 5(29.41%) | 5.39 | **0.020** |
| Living with family (N, %) ^b^ | 110(90.91%) | 93(89.42%) | 17(100%) | 1.98 | 0.160 |
| Comorbid substance abuse (N, %) ^b^ | 6(4.96%) | 4(3.85%) | 2(11.76%) | 1.94 | 0.163 |
| Age of disease onset (N, %) ^b,c^ |  |  |  | 1.81 | 0.178 |
| Early onset (≤ 25) | 53(43.80%) | 43(41.35%) | 10(58.82%) |  |  |
| Late onset (> 25) | 68(56.20%) | 61(58.65%) | 7(41.18%) |  |  |
| Duration of undiagnosed bipolar disorder (Month, Median and quartiles) ^a^ | 20.75(1.38, 78.13) | 20.25(1.06, 75.94) | 37.25(7.00, 150.13) | -1.23 | 0.220 |
| Duration of illness (Month, Median and quartiles)^a^ | 45.00(9.00, 108.00) | 41.00(7.25, 105.75) | 75.00(16.50, 162.00) | -1.71 | 0.088 |
| Polarity of first mood episode (N, %) ^b^ |  |  |  | 2.18 | 0.140 |
| Depressive | 109(90.08%) | 92(88.46%) | 17(100%) |  |  |
| Manic/hypomanic/mixed | 12(9.92%) | 12(11.54%) | 0 |  |  |
| History of misdiagnosis |  |  |  |  |  |
| Major depressive disorder (N, %)^a^ | 98(80.99%) | 86(82.69%) | 12(70.59%) | 1.39 | 0.238 |
| Schizophrenia (N, %)^a^ | 4(3.31%) | 1(0.96%) | 3(17.65%) | 12.73 | **<0.001** |
| Other mental disorders (N, %)^a^ | 19(15.70%) | 17(16.35%) | 2(11.76%) | 0.23 | 0.630 |
| Family history of mental disorders (N, %)^b^ | 41(33.88%) | 38(36.54%) | 3(17.65%) | 2.33 | 0.127 |
| History of psychiatric hospitalization (N, %)^b^ | 78(64.46%) | 64(61.54%) | 14(82.35%) | 2.76 | 0.096 |
| Lifetime suicide behavior (N, %)^b^ | 15(12.40%) | 12(11.54%) | 3(17.64%) | 0.50 | 0.479 |
| Received drug treatment within the past 12 months | 116(95.87%) | 99(95.19%) | 17(100%) | 0.853 | 0.356 |
| Antidepressants use (N, %)^b^ | 66(54.55%) | 60(57.69%) | 6(35.29%) | 2.96 | 0.086 |
| Antipsychotics use (N, %)^b^ | 77(63.64%) | 61(58.65%) | 16(94.12%) | 17.65 | **0.005** |
| Mood stabilizer use (N, %)^b^ | 100(82.64%) | 84(80.77%) | 16(94.12%) | 1.82 | 0.178 |
| Two or more mood stabilizers use (N, %)^b^ | 23(19.01%) | 19(18.27%) | 4(23.53%) | 0.26 | 0.608 |
| Received non-drug treatment within the past 12 months (N, %)^b^ | 18(14.88%) | 13(12.50%) | 5(29.41%) | 3.30 | 0.069 |
| Combined with chronic diseases requiring long-term treatment (N, %)^b^ | 12(9.92%) | 7(6.73%) | 5(29.41%) | 8.41 | **0.004** |

Note: BD P-: The patients who were without psychotic symptoms; BD P+: The patients who were with psychotic symptoms

^a^ These variables were compared by using Mann-Whitney U test

^b^ These variables were compared by using chi-square test

^c^ Following previous studies ^[38, 39]^, ﻿age of 25 years was used as the cutoff point to define early onset and late onset

Table S4. Multivariate logistic regression analysis of bipolar II disorder with psychotic symptoms.

| Independent variables | Beta | SE | Wald's test | *P* | ﻿Odds ratio | 95% Confidence interval | |
| --- | --- | --- | --- | --- | --- | --- | --- |
|  |  |  |  |  |  | Lower limit | Higher limit |
| Age | 0.06 | 0.06 | 0.95 | 0.330 | 1.06 | 0.94 | 1.20 |
| Male | -1.36 | 1.27 | 1.14 | 0.286 | 0.26 | 0.02 | 3.12 |
| Years of education | 0.38 | 0.20 | 3.78 | 0.052 | 1.47 | 1.00 | 2.16 |
| Unemployment | 1.47 | 1.27 | 1.34 | 0.248 | 4.36 | 0.36 | 52.845 |
| Living alone | -23.82 | 8605.10 | < 0.001 | 1.000 | < 0.001 | < 0.001 | - |
| Early onset | 1.14 | 1.62 | 0.49 | 0.483 | 3.12 | 0.13 | 75.01 |
| Duration of illness > 60 months^a^ | 1.86 | 1.70 | 1.19 | 0.275 | 6.43 | 0.23 | 181.07 |
| Polarity of first mood episode was manic/hypomanic/mixed | -20.50 | 8908.59 | < 0.001 | 1.000 | < 0.001 | < 0.001 | - |
| History of misdiagnosed as major depressive disorder | -0.58 | 1.29 | 0.20 | 0.656 | 0.56 | 0.05 | 7.09 |
| History of misdiagnosed as schizophrenia | 0.36 | 1.89 | 0.04 | 0.850 | 1.430 | 0.04 | 57.68 |
| History of misdiagnosed as other mental disorders | -1.14 | 1.48 | 0.59 | 0.443 | 0.32 | 0.02 | 5.85 |
| Family history of mental disorders | -2.80 | 1.53 | 3.35 | 0.067 | 0.06 | 0.003 | 1.22 |
| History of psychiatric hospitalization | -0.17 | 1.17 | 0.02 | 0.883 | 0.84 | 0.09 | 8.32 |
| Lifetime suicide behavior | -0.22 | 1.40 | 0.03 | 0.874 | 0.80 | 0.05 | 12.46 |
| Received drug treatment within the past 12 months | 10.24 | 14158.82 | < 0.001 | 1.000 | 27881.30 | < 0.001 | - |
| Antidepressants use | -1.30 | 1.15 | 1.30 | 0.255 | 0.27 | 0.03 | 2.56 |
| Antipsychotics use | 2.98 | 1.59 | 3.52 | 0.061 | 19.67 | 0.87 | 443.07 |
| Mood stabilizer use | 2.49 | 1.93 | 1.65 | 0.198 | 12.04 | 0.272 | 533.42 |
| Two or more mood stabilizers use | -1.94 | 1.48 | 1.72 | 0.190 | 0.14 | 0.01 | 2.61 |
| Received non-drug treatment within the past 12 months | 2.29 | 1.34 | 2.92 | 0.088 | 9.83 | 0.71 | 135.42 |
| Combined with chronic diseases requiring long-term treatment | 2.96 | 1.39 | 4.52 | **0.033** | 19.38 | 1.26 | 297.63 |

^a^ Following previous studies^[40]^, 60 months (i.e. 5 years) was used as the cutoff point to define short- and long- duration illness
